# Exploring the impact of health worker strikes on maternal and child health in a Kenyan county

**DOI:** 10.1186/s12913-022-08493-2

**Published:** 2022-09-09

**Authors:** Abdu Mohiddin, Eva Langat, James Orwa, Violet Naanyu, Marleen Temmerman

**Affiliations:** 1 Centre of Excellence in Women and Child Health, Aga Khan University, P.O. Box 30270-00100, Nairobi, Kenya; 2 Department of Health, County Government of Kilifi, Kilifi, Kenya; 3 Department of Population Health, Aga Khan University, Nairobi, Kenya

**Keywords:** Strikes, Maternal health, Child health, Health services research, Public health

## Abstract

**Background:**

Studies of the impact of health care workers’ strikes tend to look at facility-level activity rather than populations, with evidence from low and middle-income countries relatively sparse. This study explored the effect of national strikes on maternal and child health. It looked at the impact on health system activity in both public and non-public sectors (e.g. private, faith-based), on health promotion investments like immunisation, and on disease detection like post-partum haemorrhage (PPH). A 100 day doctors’ strike started in December 2016, a 150 day nurses strike from June 2017 and then the clinical officers for 21 days that September.

**Methods:**

Time series descriptive analysis of attendance data from the Kenyan Health Management Information System (public, non-public sector facilities). The setting was Kilifi, a coastal county in Kenya with a population of about 1.5 million.

**Results:**

Along the care pathway from antenatal, postnatal and out-patient child health clinics, activity levels dropped markedly in the public sector with only partial compensatory increases in non-public sector activity. The number of fully immunised children fell during the nurses strike as did women seen with PPH during all strikes. These health care strikes caused significant adverse health impacts at the time and potentially inter-generationally as exemplified by the fall in antenatal haematinics supplementation and syphilis testing. Some post-strike ‘’catch-up” activity occurred, however this may have been too late in some instances.

**Conclusions:**

Policy-makers at national and county level need to ensure population health is protected at times of strikes and ideally resolve disputes without such action. Not to do so risks major negative effects on maternal and child health. Increased use of the non-public health sector could be done by the authorities in mitigation should strikes occur again.

## Background

It is important to know the effects of healthcare worker (HCW) strikes especially on vulnerable populations such as pregnant women and children so that they can be averted or mitigated [1]. The impacts of strikes on health in general have been found to be on the direct provision of services [2], the healthcare system itself [3], detection of disease or complications [4] and on health promotion investments for future health [5]. The disputing parties and in particular policy-makers should be as cognisant of these impacts as possible, yet studies thus far tend to look at hospital facility level and so may not fully capture impacts at population level let alone subgroups within this. Further, studies from low and middle income countries (LMICs) are relatively sparse and so the potential differential impact on less affluent societies populations is under-estimated with attendant health system resilience shortcomings [1, 6, 7].

The 2017 strike by HCWs in Kenya therefore provided an opportunity to learn more. It involved three consecutive cadres - doctors, nurses, and clinical officers (mid-level cadre of non-physician clinicians). The strikes were about better working conditions in the public health facilities including improvement of the public health facilities and better pay and conditions. Kenyan law recognises the right to strike but requires that this does not undermine the life and health of citizens seeking healthcare [8, 9]. The strike ended with collective bargaining agreements made with the Kenyan government.

Several quantitative studies have looked at this Kenyan strike with a range of findings. Worse mortality and morbidity were seen in paediatric and obstetric in-patients in a faith-based hospital [10]. Yet another study noted differences between striking (mortality decreased) versus non-striking facilities (increased mortality), likely due to ill patients seeking help in the non-striking facilities [11] Also, reductions in paediatric admissions in public hospitals [6] and in childhood immunisations by public facilities but a rise in those by faith-based facilities [12]. Findings from a qualitative study reported limited evidence of health system preparedness for the future, major negative health and financial strike impacts on local communities, and especially the poor, and increases in private sector facility fees [13].

These studies reinforce the need for a deeper understanding of the overall impact of strikes ideally at a level that constitutes a population and its governing authority, such that public health and non-public health sector impacts and policies can be accounted for including any post-strike “catch-up” mitigation activity. This would allow an overall net picture to emerge, one that includes the potential life-course implications of lost or deferred health service activities [14].

With that in mind, studying the strike effects on pregnant women and young children is potentially insightful. They are amongst the most vulnerable groups in a population and particularly so in situations of health service stress or crisis, a reality long recognised by the UN’s Declaration of Human Rights [15]. The care pathway from pregnancy to post-natal and early childhood encompasses a range of health service interventions to care for, protect and promote the health of mother, baby and child – as such this pathway provides an opportunity to assess HCW strike impacts. The interventions that could be affected include:

- Health system activity:
  - ◦ Routine appointments are important exemplified by antenatal care which is effective at preventing and mitigating any difficulties at birth and reduces the risk of low birthweight babies, infant mortality and stunting [16]. Important here is reducing any lifestyle risks such as smoking, poor nutrition and anaemia
- Detecting disease and management:
  - ◦ Post-partum haemorrhage (PPH) is one important example as it is the cause of 30-50% or maternal mortality globally, yet can be stopped if treated promptly after childbirth [17]
- Health protection/promotion:
  - ◦ childhood immunisations are highly effective in protecting health [18]

In this paper, we explore the impact of the 2017/18 HCW strike on maternal and child health (MCH) in a county in Kenya.

## Methods

### The setting: Kilifi county and the strike

The study was done in Kilifi County, Kenya in a partnership between the Aga Khan University (AKU) and the Department of Health, Kilifi County. Counties are semi-autonomous local governments in Kenya with devolved responsibility for health service delivery. Kilifi’s health system comprises about 150 public facilities and 202 Faith/Non-governmental organisations and private facilities with most people using the public ones. [19] Kilifi is a mainly rural area on the Kenyan Coast with a population of about 1.5m in 2019 [20]. Approximately 52% of the population are female and 47% are 15 years of age and below. The main economic activities are subsistence farming and fishing, and the average monthly income per person is roughly US$8. Some 55% of the population in the County are of low socioeconomic status, with 62% of the population said to have low literacy levels.

### Strike overview

In 2016/17, Kenya experienced more than three consecutive health care workers strikes - doctors, nurses, and clinical officers. In early December 2016 to mid-March 2017, both junior and senior doctors went on 100 consecutive days strike. The nurses then followed by a 5-month ongoing strike from early June and were later joined by the clinical officers in September for a period of 21 days.

Services continued in part based on the availability of non-striking staff and this varied by facility. Level 2 (dispensaries including ANC and PNC) and Level 3 facilities (health centres with maternity in-patient care) were the most affected with larger county hospitals less so. The strike ended with a Govt led CBA with the striking workers and Counties.

### Description of the data used

Routine data were extracted from the Kenya Government District Health Information System following additional efforts by the Kilifi County Information Office to ensure the data were updated to account for any gaps in reporting and cleaned. Data collection in Kenya is mainly manual using paper based registers and tally sheets to record patient level data which are then summarised monthly in standard summary sheets. The data were aggregated for Kilifi County from the Ministry of Health tools 515 (Community Health Workers summary), 711 (MCH summary), 717 (hospital outpatient summary) which incorporate government and private and faith sector reporting and are mandatory. The time-frame covered was from December 2016 to December 2018 including these specific periods:

- Pre-strike matched period: 5^th^ December 2015 to 1^st^ November 2016
- Strike period: 5^th^ December 2016 to 1^st^ November 2017
- Post-strike matched period: : 5^th^ December 2017 to 1^st^ November 2018

Descriptive analysis was used to show the time trends in maternal and child health service activity data before, during and after the strike using Statistics and Data version 12 (STATA) [21].

### Data completeness

We looked at the data reported from facilities with services that were operational i.e. open for public use so as to ascertain those that reported activity and those that didn’t. Rather than adjust for any missing data we report the actual pre-strike figures which provide a baseline from which the additional strike impact can be estimated. In addition to the strike, their reasons for non-reporting are stock outs of commodities or no clients came for the services.

## Results

### Health system activity

**Fig. 1:**
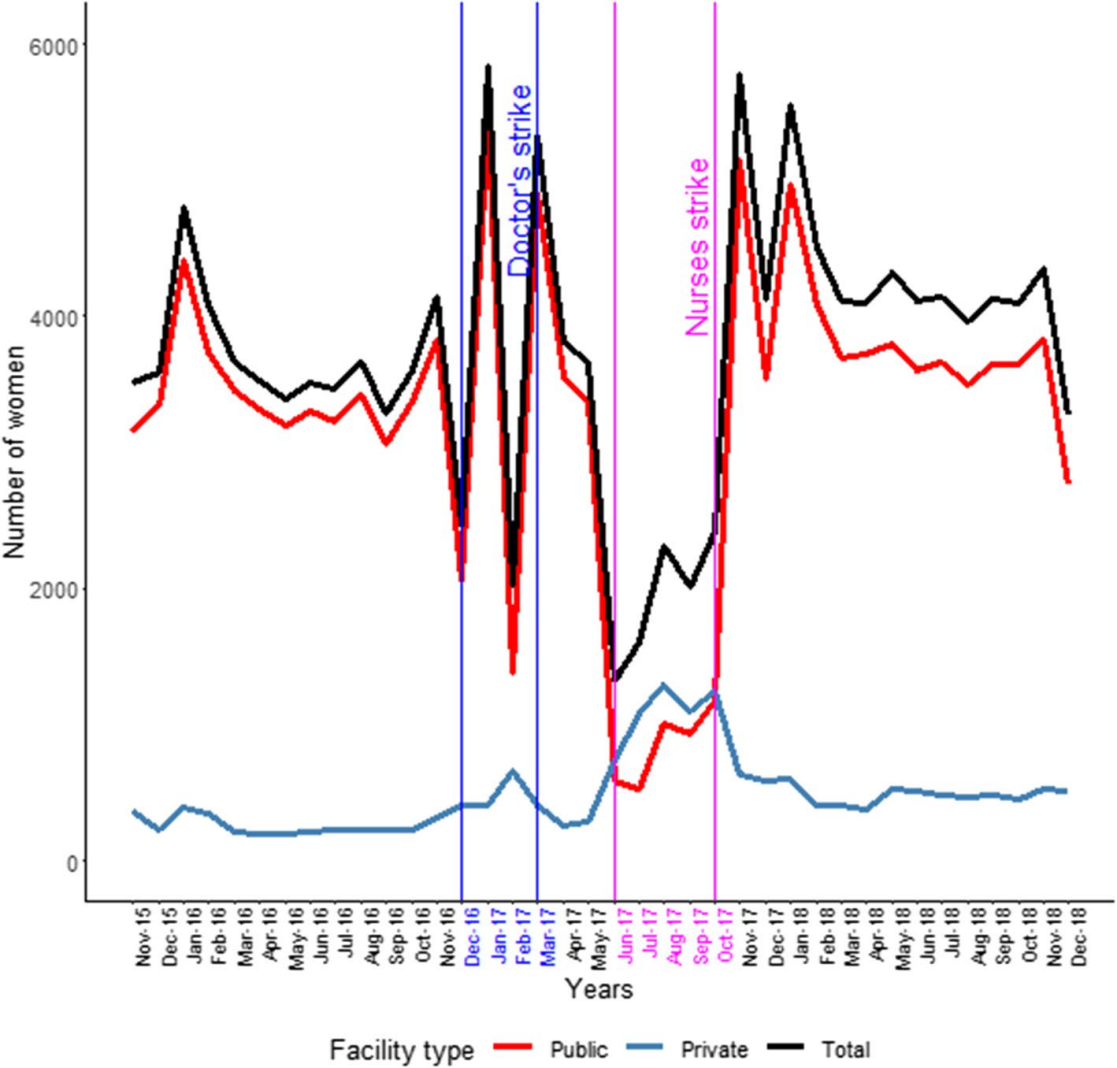
New antenatal clinic attendance

There were considerable reductions in activity along the pathway from antenatal care (ANC) to deliveries themselves and post-natal clinics (PNCs). For new ANC attendances, Fig. 1 shows this with the nurses strike being particularly effective with public facility activity very low and the accompanying increase in private facility activity far too low for there to be any net compensation. The data on four ANC attendances show a similar picture.

The ANC data completeness showed that the public sector facilities pre-strike reporting figure was 96% which decreased to 71% during the strike. For the non-public sector facilities, the figures were 65% and 71% respectively showing that reporting increased most likely due to new facilities opening up.

**Fig. 2:**
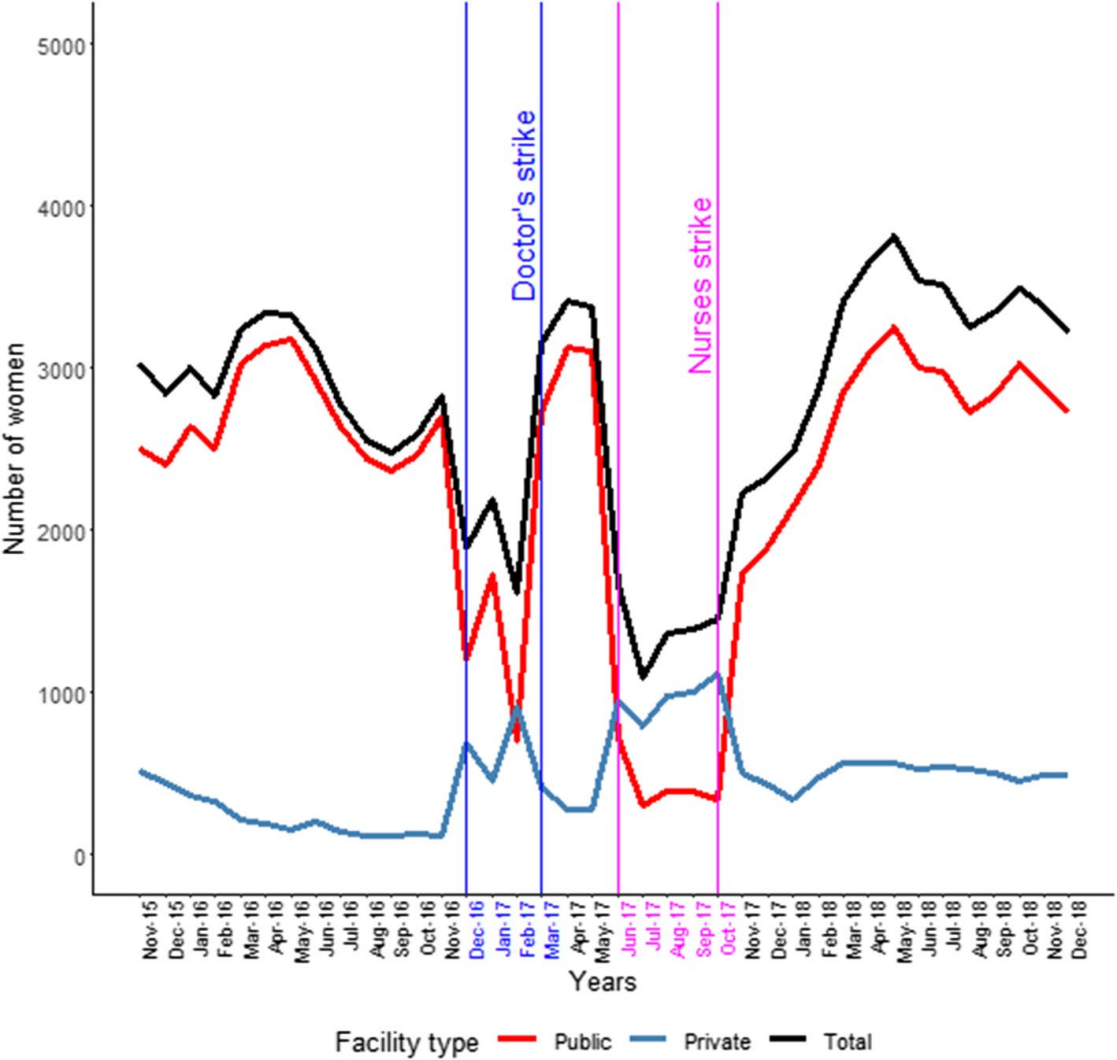
Total deliveries

The total number of deliveries in the County are in Fig. 2 and this shows how the doctors and nurses strikes both had an effect with the public facility impact greater for the latter cadre. Private facility activity increased but the net effect was a reduction.

For deliveries, data completeness showed that 81% of public sector facilities reported activity pre-strike which declined to 54% during the strike, for non-public sector the figures were 42% to 55%, again due to new facilities opening up.

**Fig. 3:**
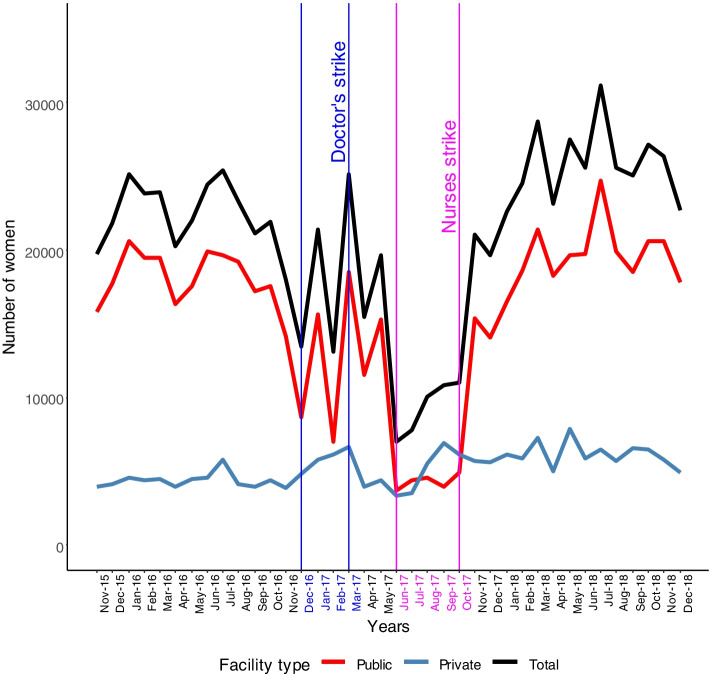
Post-natal clinic NC attendance

For the PNCs the overall net effect was a reduction in activity, more so with the nurses’ strike (Fig. 3).

For PNC, data completeness was 49% of public sector facilities reporting activity pre-strike which declined to 30% during the strike, for non-public sector figures were 9% to 9%.

### Health promotion investments

Promoting better health and protecting existing health are integral parts of the care pathway and these activities were also affected.

Antenatally, the data on haematinics supplementation (folic acid, iron) were similar to those in Fig. 1, i.e. an overall net reduction of at least two-thirds was seen with the nurses strike as the main contributor to this decline and very small increases in private facility activity.

**Fig. 4:**
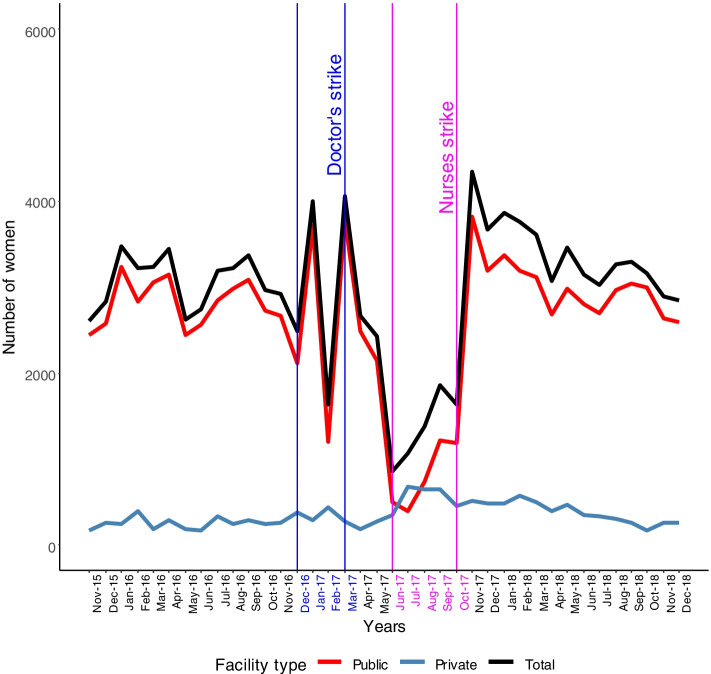
Fully immunised children

Childhood immunisations were adversely affected too with Fig. 4 demonstrating the data on fully immunised children i.e. received all the immunisations and at least one Vitamin A dose under the national schedule before the first birthday. There was a significant decline particular during the nurses strike with a modest private facility increase and a suggestion of a post-strike “catch-up” increase in immunisation activity.

For fully immunized children, data completeness was 93% of public sector facilities reporting activity pre-strike which declined to 67% during the strike, for non-public sector figures were 59% to 51%.

### Detecting disease and management

**Fig. 5:**
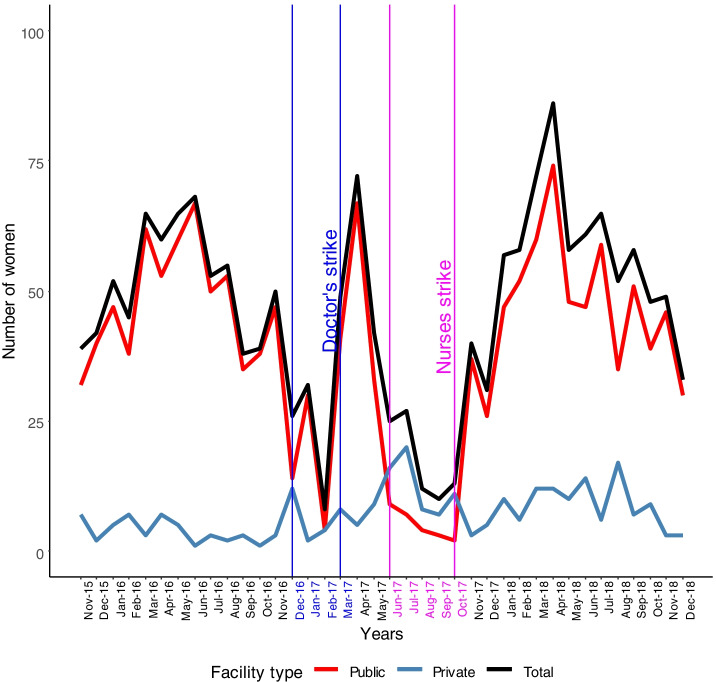
Post-partum haemorrhage

Testing for syphilis is routinely done on all pregnant women and whilst there was an overall net reduction of about half, private facility activity rose from a low level and approximately doubled. Access to Caesarean sections also had a net decline with both doctors’ and nurses’ strikes contributing to this accompanied by about three-fold rises in private facility activity. PPH activity is shown in Fig. 5 with net reductions during both the doctors’ and nurses’ strikes and small increases in private sector activity.

For PPH, only 9% of public sector facilities reported activity pre-strike which declined to 5% during the strike, for non-public sector figures were 1% to 2%.

## Discussion

Overall, this exploratory study has highlighted the substantial negative effect of the strike on MCH in Kilifi, both on the then clients of services and the next generation too – a lifecourse double whammy.

Along the MCH pathway significant net shortfalls in activity suggested that many clients were left to the mercy of their own resources, whether it be seeking non-public facility or traditional help such as birth attendants. This is most likely to have worsened inequalities with many poorer mothers bearing a higher risk of worse outcomes for themselves and their babies. It is highly probable that Kilifi County has had more low birth weight, neural tube defected and syphilitic babies, and children with preventable infectious diseases to care for than otherwise. It also has most probably suffered greater maternal and newborn mortality – the decline in PPH attendances alone points to this though reporting was low in general, in common with that seen in the Kenyan health system. The data show that there were some post-strike “catch-up” activities which demonstrates how local authorities can attempt to mitigate strike impacts.

A Danish study of a national nurses’ strike showed that strike exposed mothers were less likely to breastfeed children at four months [22]. The study also described a substitution effect of nursing care by family doctors which helped blunt the overall effect. In our Kilifi study the only similar substitution-type effect seen was through the private sector facility activity albeit this was insufficient. Traditional health care may have helped fill the gap but data on this were not available. It is difficult to identify the precise contributions of the differing HCW cadres especially for the clinical officers as their strike coincided with the end of the nurses’ one and was shorter. But of the three cadres of staff involved – nurses, doctors and clinical officers – it appears that the nurses strike had the greatest impact which has some resonance as for much of the everyday MCH care that is required, nurses were critical and did most of the routine healthcare. Nurses are the backbone of MCH care, are critical along the whole MCH care pathway and deliver care in the lowest level of the health care system including in rural areas where most people live.

The considerable length of this Kenyan strike is relevant too potentially more so for children. Friedman et al [23] looked at strikes in Kenya from 1995-2014 and combined retrospective data on births with data on strikes, finding 32 strikes over 11 counties and 16 hospitals. Their results suggested that children born during strikes were more likely to die, less likely to be fully vaccinated and have lower height and weight scores in their first year even though most of the strikes were in single facilities and short-lived (one week typically). A study of many strikes by health workers in Kilifi from 2010-16 (median length 18.5 days) suggested no change in all-cause mortality with weak evidence of a decrease in mortality in children aged 1-11 months but an increase in those 1-5 years old [24]. Our study emphasises the view that MCH are especially vulnerable.

By taking this more specific look at MCH in a local county, this study has been able to further investigate the strike effects rather than have the impact on this group subsumed in more general findings of facility-level all-age mortality or morbidity data. The evidence base of studies using this MCH approach to strikes is very limited and presents a clear research gap within which the health and socioeconomic impacts are potentially huge. Future research could use epidemiological and economic modelling to further assess the impact of the strike using local or published estimates of morbidity and mortality rates.

Finally, there is a need for governments (local and national) and other stakeholders to recognise these significant impacts of strikes on MCH and work to prevent strikes, protect health through minimal core services (including emergencies) and mitigate post-strike. In this effort, using all local resources including the non-public sector is essential [25]. Integral to this is understanding the lifecourse and the effects of any disruption especially as the law in Kenya states that strikes should not undermine health [26]. So there is a need for advocacy, raising rights awareness and seeking redress for the many who have had their health undermined [27].

There are some limitations of this study. It has taken an exploratory approach to the strike impact question and as such provides a basis for future studies – it points to this need for more research especially the equity and justice aspect of bringing together population and intergenerational impact with its accountable governing institution. Looking at a county level plays to the devolved nature of health policy in Kenya but a similar study to this one could be done at national level too. Attempts were made to have the health information system data as clean and complete as possible especially from the non-public sector facilities. There were several possible indicators to look at and we chose the ones we did to better represent the important points of care along the pathway from antenatal to birth so as to have a more comprehensive look at this pathway impact of the strike. Whilst this also comes at the cost of more detailed individual indicator analysis, the hope is that it is helpful in exposing the overall detrimental effect of the strike. However, there may still be shortcomings in this data completeness, we did not impute or adjust for missing data, we reported what was recorded and believe that this in the main reflects the effect of the strike on activity and is a realistic picture of what county health officials would have to understand their situation and inform action. But it could also be due to other more everyday data completeness issues like simply not being reported. The non-public sector providers report less reliably than the public sector but the County does make efforts to improve this and so an under-estimate of this sector’s activity is possible - interestingly the reported data showed that the sector did scale up its provision relatively quickly during the strike in response to need. Also, we did not collate data from other counties and so any Kilifi clients who opted to use facilities in neighbouring counties and vice versa were not counted. Given the nature of this aggregate data, individual-level characteristics could not be considered. We have associated changes in activity levels to outcome changes by referring to studies that demonstrate that e.g. immunisations. Finally, the indicators we used reflected more nurse-led activity then the other cadres.

## Conclusion

This study explored the impact of HCW strikes on MCH in a local area. Activity levels along the MCH pathway were adversely affected overall with large declines in public facility activity only partly offset by increases in the private and faith-based sector. Nurse-led activities appear to have been worst hit of all the cadres involved. Overall, these impacts are likely to have widened health inequalities in mortality and morbidity for the mothers and have negative lifecourse consequences for their newborns and children. Governments and other stakeholders should take note of these impacts to ensure that strikes are prevented, have protection of health arranged should they occur with post-strike mitigation actions too.

## Data Availability

The data are available from the Kenyan Health Management Information System (KHIS). All data relevant to the study are included in the article. Contact information: Abdu Mohiddin; email: abdu.mohiddin@aku.edu
